# Editorial: The role of emotion regulation on the developmental course of eating disorders, obesity and food addiction

**DOI:** 10.3389/fpsyt.2024.1436479

**Published:** 2024-06-10

**Authors:** Roser Granero, Isabel Krug, Geovanny Genaro Reivan Ortiz

**Affiliations:** ^1^ Department of Psychobiology and Methodology, Universitat Autònoma de Barcelona, Barcelona, Spain; ^2^ Melbourne School of Psychological Sciences, The University of Melbourne, Melbourne, VIC, Australia; ^3^ Eating Behavior & Methodology Laboratory (EM-LAB), Catholic University of Cuenca, Cuenca, Ecuador

**Keywords:** emotion regulation, eating disorder, food addiction, obesity, psychopathology

## 1 Emotion regulation: a complex transdiagnostic construct

Emotion regulation (ER) is a widespread topic across many areas within psychology, which includes a set of strategies aimed to identify and manage the course of the individuals’ emotions (1). Interrelated processes implicit in this concept (both automated and controlled mechanisms) are focused on coping with the internal experiences and the external expressions when emotional states interfere with concrete desired achievements (2). Key components of ER are the ability to regulate abilities such as impulsivity, planning, coping skills and frustration tolerance (3).

Diverse conceptualizations of ER exist, which differ in different Research Topics (4): a) the differentiation between the components of emotions and the processes of regulation; b) the focus on the explicit versus explicit nature of ER; and c) the center on intrapersonal versus interpersonal processes. One of the theoretical models for ER most accepted within applied clinical research and treatment contexts is the *Multidimensional Process of Emotion Regulation* (5), which hypothesizes an adaptive response process to emotional distress which is structured in five broad facets of emotion regulation: 1) lack of emotional awareness; 2) lack of acceptance of emotions; 3) difficulty in controlling impulses and behaviors in the presence of negative affect; 4) lack of clarity of emotional experiences; and 5) difficulties to access to emotion regulation strategies. An other model with high acceptance in the clinical literature is the *Gross’s Process Model of Emotion Regulation*, which supports that individuals activate and employ a number of ER mechanisms (6): a) selection/modification of situations to decrease negative impact on the emotional states; b) selective attention on concrete features of the situations; c) choosing the more beneficial emotional meaning of the situations; and d) modulating one’s emotional response once a certain emotion has been generated. Two reasons for the wide acceptance of these two models, in contrast with alternative emotion-based frameworks, is the broad conceptualization of ER (into a multicomponent schema) and the focalization on the trail-level abilities (instead of processes).

Adequate ER provides suitable functional resources and appropriate coping strategies in stressful situations, and therefore an adept ER is a protective factor for health and well-being. In contrast, maladaptive ER strategies (such as rumination, avoidance, and suppression) are strongly associated with the onset and the progression of mental disorders, as far as to propose that ER could be conceptualized as a *transdiagnostic* mechanism underlying psychopathology (7, 8). In accordance with this notion, disordered eating behaviors often resulting in severe clinical eating disorders could be the result of dysfunctional strategies used to avoid/suppress negative emotional states (stress, anger, sadness, fear, anxiety, or depression) (9). Consequently, patients with eating related problems often present with not just the severe disordered eating symptoms (binge eating and/or weight-control behaviors [restrictive food intake, vomits, excessive exercise, and use of laxatives and diuretics], but also difficulties in ER and comorbid psychological distress (10).

More recently, the conceptualization of ER as a transdiagnostic mechanism for mental illness has been the basis for the development of psychological interventions that target ER (11, 12). Such intervention studies explored how improvements in ER capacities resulted in increases in global psychological wellbeing or prevented the emergence of full-blown mental health disorders (13, 14). Besides the popularity of these transdiagnostic interventions there is currently no agreement regarding what aspects of this intervention target which ER dimension and what the longitudinal effects of these interventions are. Finally, possible mediating variables (such as sex or age) that could fully or partially explain the relationship between the different ER dimensions and mental health have not been fully investigated. However, these mediational relationships may be important to further refine the transdiagnostic interventions for ER and mental health. (15, 16).

## 2 Motivation for this Research Topic

This Research Topic is focused on the study of the structure of the different processes underlying ER and its specific contribution on dysfunctional eating patterns in the community and clinical populations. Highlights of the special include: a) exploring the underlying mechanisms (and pathways) between the multiple domains of ER and unhealthy eating profiles; b) identifying the interrelations between ER with other psychological and biological markers related with the onset and the developmental course of eating problems; c) assessing the specific predictive capacity of the ER strategies on the treatment outcomes planed for impaired eating behaviors; d) identifying empirical developmental trajectories (and latent clusters) based on ER of specific populations suffering from disordered eating symptoms (e.g. eating disorders, obesity, food addiction).

## 3 Aims and empirical evidence of the manuscripts included in the special

Some of the studies included in our Research Topic analyzed the potential role of ER in the etiology of eating related problems. First, the research published by Law et al. for instance is an umbrella review aimed at summarizing multiple mental health outcomes (e.g. anxiety and depression symptoms) among patients with bariatric surgery. The review concluded that this surgery is beneficial in improving overall mental health. However, the authors also remark the increased risk of suicide and self-harm behaviors during the follow-up period after the surgery, that could be the consequence of pre-existing mental illnesses and/or the result of potential postoperative problems related with ER difficulties (such as difficulty controlling pain or scarring). Another study included in this Research Topic assessed different characteristic of eating behaviors and factors related with grazing behavior during the preoperative period of bariatric surgery (Kikuchi et al.). This cross-sectional research analyzed data recruited among obese patients attended at a reference treatment unit in a public hospital and observed that the use of weight loss drugs correlated with increased cognitive restriction, and lower levels in uncontrolled eating and grazing.

The cross-sectional study by Reivan Ortiz et al. assessed in a population-based sample a predictive model of perceived stress during COVID-19 and explored the mediational links (Reivan Ortiz et al.). This work considered a large set of variables including ER, perceived stress, active procrastination, social isolation, and other sociodemographic and psychological symptoms (including problematic eating behaviors). Another study by the same research team also included in this Research Topic was aimed to obtain evidence about the underlying processes explaining the presence of food addiction, considering a large set of indicators (eating-related measures, impulsivity, psychopathology state, and ER) (Reivan Ortiz et al.). In this work, path analysis methodology suggested that being women, older age, more difficulties in ER, higher impulsivity levels, negative mood and anxiety triggered disordered eating. It was also observed that higher impairing in the eating behavior correlated with increased food addiction level.

The authors Momeñe et al., a cross-sectional community research, examined the relationships between ER strategies with self-image and body dissatisfaction. Authors concluded that anger states impacted on body dissatisfaction, non-acceptance of emotional responses, lack of emotional awareness and lack of emotional clarity; additionally, impulse control deficit was the ER factor with the highest contribution on the body dissatisfaction, and this was an indirect relationship mediated by increased anger.

The authors Téllez-Rodríguez et al. observed that higher food addiction levels were associated with more impaired executive functioning, greater reward sensitivity, more severity in depression symptoms and higher number of binge eating problems. Authors also outlined that decreased crystallized intellectual capacity and impaired control food intake were linked to higher body mass index for participants with higher number of food addiction symptoms.

Two studies included in this Research Topic provide evidence regarding impact of ER in eating disorders profiles and treatment outcomes. Martinelli et al. analyzed impulsivity and reward and punishment sensitivity on eating related problems, hospital course and treatment results, in a sample of patients admitted to a specialized inpatient behavioral therapy (Martinelli et al.). The authors concluded that impulsivity domains may help discriminate restrictive versus binge/purge problematic eating, but it has an unclear role in the inpatient program.

Finally, the authors Dol et al. assessed the relationships between the BMI, emotional eating and emotional stability, and its potential predictive value on the individuals’ identification with the problem. The results of this work may offer perspective for the development of personalized virtual coach applications designed to emotional eating habits. Authors outline that these interventions should be based on dialectical coaching strategies as preferred by participants, tailored to the individual ER skills and the concrete stages of awareness of their emotions.

## 4 Implications and future directions

Assessing ER may have clinical utility as a measure of the global psychological wellbeing. The identification of ER difficulties is also a potential prognostic index of the individual’s mental health problems, and therefore special attention should be given to the patients’ behavior in daily functioning, particularly in stress situations. The results obtained in the studies included in this Research Topic could contribute to a better comprehension of the specific associations between ER with impaired eating behaviors. The findings of the studies could also lead to more effective prevention and treatment modalities, including multiple variables that can contribute to successful responses (in addition to the core eating related symptoms).

## Author contributions

RG: Writing – original draft, Writing – review & editing. IK: Writing – review & editing. GRO: Writing – review & editing.
